# Biosynthesis, Spectrophotometric Follow-Up, Characterization, and Variable Antimicrobial Activities of Ag Nanoparticles Prepared by Edible Macrofungi

**DOI:** 10.3390/biom13071102

**Published:** 2023-07-11

**Authors:** Mohamed S. Youssef, Sanaa Ibrahim Ahmed, Ibrahim M. A. Mohamed, Marwa M. Abdel-Kareem

**Affiliations:** 1 Botany and Microbiology Department, Faculty of Science, Sohag University, Sohag 82524, Egypt; youssefm2006@yahoo.com (M.S.Y.); sanaaibrahim1989@gmail.com (S.I.A.); marwaabdelkareem7@gmail.com (M.M.A.-K.); 2 Chemistry Department, Faculty of Science, Sohag University, Sohag 82524, Egypt

**Keywords:** silver nanoparticles, *Pleurotus floridanus*, spectrophotometric follow-up, antimicrobial agent, biosynthesis

## Abstract

The biosynthesis of silver nanoparticles (Ag NPs) could play a significant role in the development of commercial antimicrobials. Herein, the biosynthesis of Ag NPs was studied using the edible mushroom *Pleurotus floridanus,* and following its formation, spectrophotometry was used to detect the best mushroom content, pH, temperature, and silver concentration. After that, the morphology was described via transmission electron microscopy (TEM), and nanoscale-size particles were found ranging from 11 to 13 nm. The best conditions of Ag content and pH were found at 1.0 mM and 11.0, respectively. In addition, the best mushroom extract concentration was found at 30 g/L. According to XRD analysis, the crystal structure of the formed amorphous Ag NPs is cubic with a space group of fm-3m and a space group number of 225. After that, the function groups at the surface of the prepared Ag NPs were studied via FTIR analysis, which indicated the presence of C=O, C-H, and O-H groups. These groups could indicate the presence of mushroom traces in the Ag NPs, which was confirmed via the amorphous characteristics of Ag NPs from the XRD analysis. The prepared Ag NPs have a high impact against different microorganisms, which could be attributed to the ability of Ag NPs to penetrate the cell bacterial wall.

## 1. Introduction

Given the medical concerns related to the antibacterial resistance and biocompatibility of commercial antimicrobials, there is considerable interest in materials with a high antimicrobial behavior that could be obtained from edible foods [1,2]. Among edible foods, mushrooms attracted many researchers in different applications, including food packaging [3], pharmaceuticals [4], as a protein source [5], lactic acid production [6], agri-food supply [7], and as a potential prostate cancer retardant [8]. Additionally, edible mushrooms were studied for their antioxidant, antitumor, and antimicrobial qualities [9,10]. In addition, edible mushrooms are widely available worldwide compared to other precious antimicrobial agents. Therefore, the interest in using edible mushrooms for the design of a novel antimicrobial is high in recent research [11]. Mushrooms have a wide range of necessary biomolecules, including vitamins, steroids, polyphenols, polysaccharides, and amino acids [12,13,14]. These biomolecules could play a role as capping and reducing agents for the bio-fabrication of nanoparticles (NPs) [15]. Therefore, its extract could reduce the expected aggregation and so develop a new methodology for the large-scale production of NPs.

Among the reported methods to use microorganisms for the design of NPs, fungi are one of the best options for most NPs [16,17,18], such as Ag [19], ZnO [20], gold (Au) [21], selenium (Se) [22], iron (Fe) [23], nickel (Ni) [24], copper oxide [25], TiO_2_ [26], and iron-oxide [27]. From these NPs, silver nanoparticles (Ag NPs) were reported to possess excellent antibacterial [28], antifungal [29], antitumor [30], and anti-inflammatory qualities [31] and were useful in a range of different biomedical applications [32]. The biosynthesis of Ag NPs has various merits, including being low-cost, eco-friendly, and having a simple-step methodology, as well as a high level of biosafety in human therapeutic use. In contrast, the chemical methods to prepare Ag NPs using different hazardous chemicals affect their biocompatibility and biosafety. In addition, there is an expected high cost and deficiency of some utilized chemicals as reducing or capping agents. Since these chemical techniques have clear demerits, it is time to replace them with more bio-suitable techniques, such as edible fungi-assisted methodology. In this regard, with this synthetic strategy to produce Ag NPs, there are no special requirements, such as the use of high temperature, abnormal pressure, or toxic materials [33].

Up to now, a lot of reported work on having Ag NPs as antibacterial agents has been introduced to the literature [34], as well as their use in catalytic [35] or biomedical applications [36]. So far, limited reports have followed Ag NP formation, which is an important factor in the identification of the optimum conditions for the commercialization of Ag NPs in biomedical fields. This work presents the spectrophotometric follow-up of the formation of Ag NPs using the edible macrofungi-assisted methodology. The edible macrofungi act as both capping and reducing agents. The aim of this study was to introduce the best conditions to bio-fabricate Ag NPs and investigate this prepared material as an antimicrobial agent against various microorganisms, including Gram-positive (+) and Gram-negative (-) bacteria, candidiases, and fungi. In particular, the prepared Ag NPs in this study were evaluated against *Bacillus subtilis*, *Staphylococcus aureus*, *Bacillus cereus*, *Escherichia coli*, *Klebsiella pneumoniae*, *Pseudomonas aeruginosa*, *Salmonella typhi*, *Candida albicans*, *C. glabrata*, *C. stellatoidea*, *C. parapsilosis*, *Aspergillus niger*, and *Fusarium oxysporum*. Additionally, the prepared Ag NPs have been characterized by transmission electron microscopy (TEM), X-ray diffraction analysis (XRD), zeta potential, and Fourier-transform infrared spectroscopy (FTIR). The introduced Ag was tested at different concentrations to find the minimum inhibition concentration (MIC) against all previously mentioned microorganisms. Subsequently, the effects of different conditions on the biosynthesis of Ag NPs have been investigated.

## 2. Materials and Methods

### 2.1. Collection of Mushroom Spawn and Other Chemicals

Silver nitrate (AgNO_3_) was used without purification as a silver source after dissolving it in distilled water and was provided by Sigma–Aldrich. The utilized mushroom spawns of *Pleurotus floridanus* (PF strain) were purchased from the Agricultural Research Center, Cairo, Egypt. The mushroom was cultivated as described in Supplementary Material and as reported in recent studies [37,38].

### 2.2. Myco-Synthesis and Purification of Ag NPs

The mushroom extract was obtained by dissolving 30 g of fresh mushroom in 1 L of pure water after washing the cultivated mushroom with water and cutting it into small pieces, followed by soaking it overnight and then filtering using Whatman filter paper No. 1. The source of silver ions [AgNO_3_] was added to the filtrate (1 mM) to promote the formation of Ag NPs under room conditions. The silver/mushroom extraction mixture was stored at 30 °C for one day. The first indication for the formation of silver nanoparticles is the color change from colorless to brown [39,40]. The obtained Ag NP mixture was centrifuged at 15,000 rpm for 15 min. The synthesized Ag NPs were purified by washing using sterilized H_2_O to eliminate any adsorbed impurities.

### 2.3. Microorganism Source

This study used human clinical pathogens: three Gram (+) *Bacillus subtilis*; *Staphylococcus aureus* ACCB 136, and *Bacillus cereus* ACCB 135, four Gram (-) including *Escherichia coli*, *Klebsiella pneumonia* ACCB 202, *Pseudomonas aeruginosa*, and *Salmonella typhi,* and four pathogen yeasts (*Candida albicans*, *C. glabrata*, *C. stellatoidea*, and *C. parapsilosis*), and other fungus species (*Aspergillus niger* and *Fusarium oxysporum* AUMC 3191) were found in the Bacteriological Laboratory, Sohag University, Egypt.

### 2.4. Spectrophotometric Follow for the Ag NPs Formation

There are different factors that could play a significant role in NPs preparation from biosources, such as pH media, temperature, and concentration of metal substrate, in addition to the effect of biosource contents (mushroom extraction in this study) [41,42,43]. Therefore, spectrophotometric follow-up for the Ag NPs formation was studied at different AgNO_3_ concentrations, pH media, temperatures, and mushroom extraction concentrations. Each factor was studied deeply by following the highest absorption peak in UV-visible spectroscopy. Different AgNO_3_ concentrations (0.5 mM, 1.0 mM, and 2.0 mM AgNO_3_), temperatures (30 °C, 40 °C, 60 °C, 80 °C, and 100 °C), pH medium (3, 5, 7, 9, and 11), and different mushroom extraction concentrations (10 g/L, 30 g/L, 50 g/L, 70 g/L, and 100 g/L) were investigated.

### 2.5. Characterization of the Ag NPs Formation

The silver ion reduction was affirmed via spectrophotometric scan using ultraviolet (UV)-visible spectroscopy (JENWAY 7315 spectrophotometer, Staffordshire, UK) in the λ range of 300–700 nm. Transmission electron microscopy (TEM) analysis was applied to investigate the morphology and particle size of the prepared Ag NPs (TEM, Electron Microscope Unit, Assiut University, Egypt). The TEM images of the synthesized Ag NPs were captured randomly. Fourier-transform infrared (FTIR) analysis was carried out to understand the chemistry of the synthesized Ag NPs by knowing the function/chemical groups at the surface of Ag particles. FT-IR of the prepared Ag NPs was monitored by using a range from 400 to 4000 cm^−1^ (ALPHA II, with platinum ATR, Ettlingen, Germany) by the pure potassium bromide pellet method. The crystallinity nature of Ag NPs was checked by X-ray diffraction (XRD) technique and evaluated at 2θ = 30–80° at 40 keV using model D8 Advanced Bruker (λ = 1.54056 Å).

### 2.6. Antimicrobial Investigation

The antimicrobial activity of the prepared Ag NPs from mushroom extraction was assayed against Gram-positive and Gram-negative pathogens, yeast, and fungus species. The utilized bacteria were grown on nutrient agar (2.5 g NaCl, 2.5 g peptone, 1.5 g yeast, and 8.5 g agar in 500 mL H_2_O), which was saved in an autoclave and left to cool before being poured into Petri dishes. Then, these dishes were stored at 25 °C for 1 h. After that, the applied bacteria were spread onto separated agar using sterile cotton swabs. Wells were carried out on the prepared agar to permit the investigated material to interact with the applied bacteria. A total of 100 µL of the Ag NPs were used at different concentrations to find the minimum inhibition concentration (MIC) for each microorganism. Then, the formed plates were incubated at 37 °C for one day, followed by measuring the formed inhibition zones in mm. These assays were done three times to confirm the estimated inhibition zone [44,45].

## 3. Results and Discussion

In this work, Ag nanoparticles (NPs) were prepared via a simple, green, and biocompatible method using macrofungi (edible mushroom *Pleurotus floridanus*). Then, spectrophotometric follow-up was studied to find the best conditions for metal concentration, pH media, temperature, and mushroom concentration.

### 3.1. Spectrophotometric Follow-Up of Ag NPs Formation at Different AgNO_3_ Concentrations

**Figure 1:**
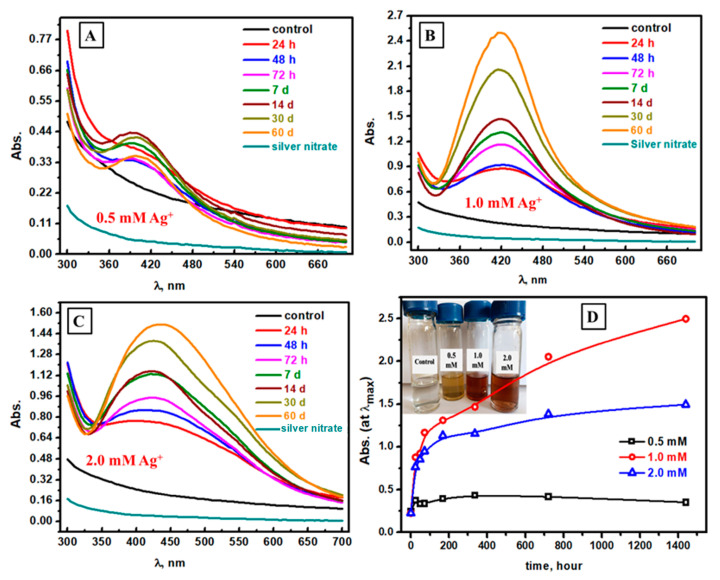
Spectrophotometric study of the formed Ag NPs at different silver ion concentrations: 0.5 mM (**A**), 1.0 mM (**B**), 2.0 mM (**C**), and visual combined with absorbance values comparison between the studied silver ion concentrations (**D**).

The optical scans of the Ag NPs formation at different reaction times from 24 h to 60 days, including the spectrophotometric scan of the mushroom substrate (control) and Ag ions, were shown in Figure 1. The fastest sign of the successful biosynthesis of Ag NPs is the change of color of the mixture (mushroom extract and silver ions) from colorless to yellowish-brown. The investigation of spectrophotometric analysis confirms what was observed by direct visual observation. The spectrophotometric study was conducted at three different silver nitrate concentrations, from 0.5 mM to 2.0 mM. Figure 1A–C displayed the spectrophotometric analysis in the presence of 0.5 mM, 1.0 mM, and 2.0 mM, respectively. The mixture of mushroom extract and silver ions has a new peak related to the successful formation of Ag NPs at λ_max_ = 420 nm [46,47] with different absorbance values according to the progressive reaction time. As the reaction time went up, the absorbance value increased up to 14 days at 0.5 mM silver ion. After 14 days, the coagulation of Ag NPs was observed in both visual and spectrophotometric analysis, which could be the main reason for absorbance decay after 14 days [48]. Interestingly, the time of Ag NPs stability sharply increased after increasing the Ag ions contents from 0.5 mM to 1.0 mM, as shown in Figure 1B. In general, the absorbance value increased up to 60 days at 1.0 mM silver ion. After that, the higher concentration of Ag ions leads to a decrease in the nanoparticle’s stability, as displayed in Figure 1C. In particular, the absorbance value increased up to 60 days at 2.0 mM silver ion, with lower absorbance values if compared with 1.0 mM silver ion. The direct comparison between the studied Ag concentrations to form Ag NPs by visual observation and absorbance values was described in Figure 1D. As seen, the best one is 1.0 mM, which has the darkest color in visual observation and the highest absorbance in spectrophotometric analysis. In short, the best AgNO_3_ molarity during the biosynthesis of Ag NPs is 1.0 mM.

### 3.2. Spectrophotometric Follow-Up of Ag NPs Formation at Different pH Values

**Figure 2:**
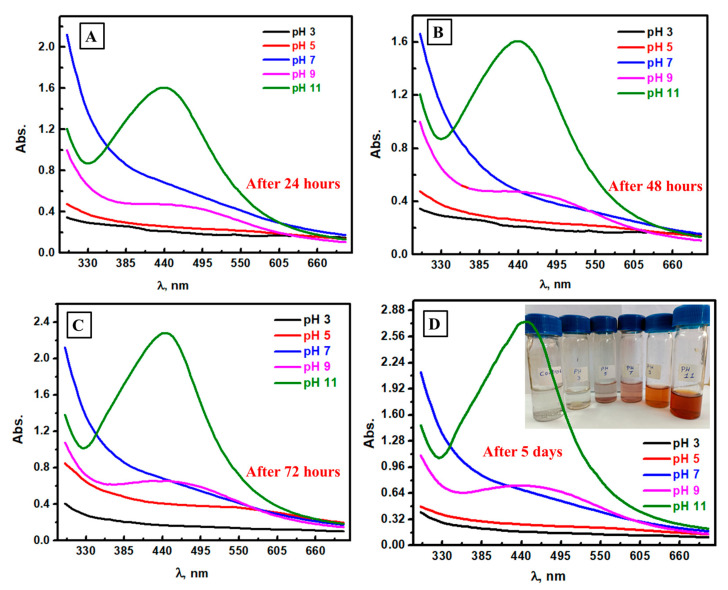
Spectrophotometric study of the formed Ag NPs at different pH media after 24 h (**A**), 48 h (**B**), 72 h (**C**), and five days (**D**).

The second factor in its effect on the biosynthesis of Ag NPs was the pH of the utilized medium. The reported optimal pH value for the preparation of Ag NPs has varied using different microbial strains [49]. Herein, different pH conditions at 3, 5, 7, 9, and 11 at different times (24 h, 48 h, 72 h, five days, and seven days) as shown in Figure 2 (Figure 2A–D and Figure S1 (Supplementary Material), respectively) were investigated in this work, which led to proving that the optimum pH could be at 11 as the clear spectrophotometric peak of Ag NPs could be seen only in the case of pH 11. Additionally, no clear color could be detected at low pH (3–7); brown color formation began at pH 9 beside the optimum one (pH 11). Consequently, the pH media should be adjusted to 11 to prepare Ag NPs. This result could be attributed to the availability of more hydroxide ions at pH 11, which could provide electrons for reducing Ag^+^ ions to Ag^0^ [45]. This conclusion agrees with earlier publications that the existence of hydroxide as a negative ion is necessary for the reduction of Ag ions [50,51,52]. In neutral media, the time required to reduce Ag^+^ ions was longer, confirming the considerable role of OH^−^ ions in reducing silver ions. An increase in alkalinity could lead to the aggregation of Ag particles. Therefore, the optimum or best pH medium is 11 in the case of the biosynthesis of Ag NPs using edible mushrooms.

### 3.3. Spectrophotometric Follow-Up of Ag NPs Formation at Different Temperatures

**Figure 3:**
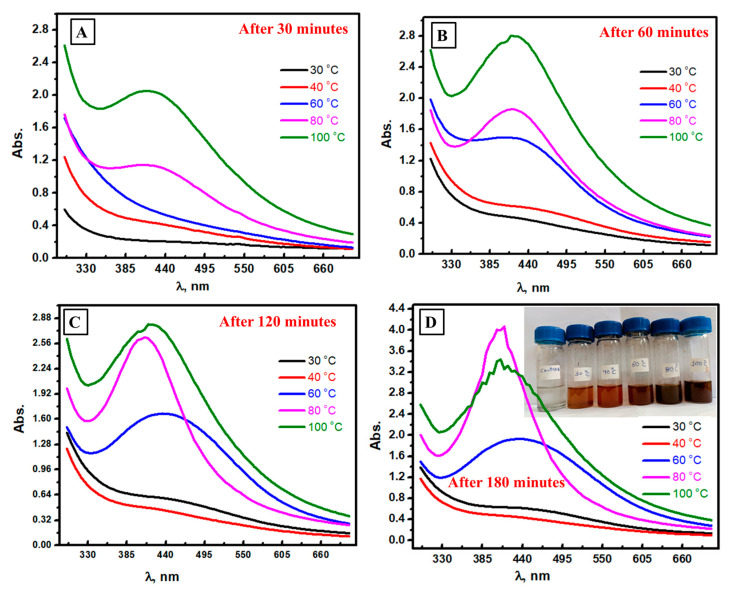
Spectrophotometric study of the formed Ag NPs at different temperatures after 30 min (**A**), 60 min (**B**), 120 min (**C**), and 180 min (**D**).

The influence of temperature on the reduction of silver ions using mushroom extract was investigated by incubating the mixture of silver ions and mushroom extract at variable temperatures (30, 40, 60, 80, and 100 °C), as shown in Figure 3A–D, respectively. After 30 min, the increasing temperature led to higher absorbance values in the range of Ag NPs absorbance, and similar results were seen after 60 and 120 min. In contrast, the scan at 100 °C starts to decrease after 180 min, which confirms the start of Ag NPs aggregation. Thus, the required time to accomplish the maximum production of silver NPs decreased as the temperature increased, as reported before [53,54]. This result could be interpreted as higher reaction kinetics associated with higher temperature conditions. Therefore, it could be expected that at lower temperatures (25 °C or 30 °C), the required reaction time for the initial synthesis of Ag NPs goes up, and the rate-determining step becomes slower, in addition to the higher mobility of silver ions at higher temperatures (80 °C and 100 °C) [55]. In short, higher temperatures lead to the formation of Ag NPs in a short time, and the required time to reduce Ag ions increases with decreasing the applied temperature.

### 3.4. Spectrophotometric Follow of Ag NPs Formation at Different Mushroom Contents

**Figure 4:**
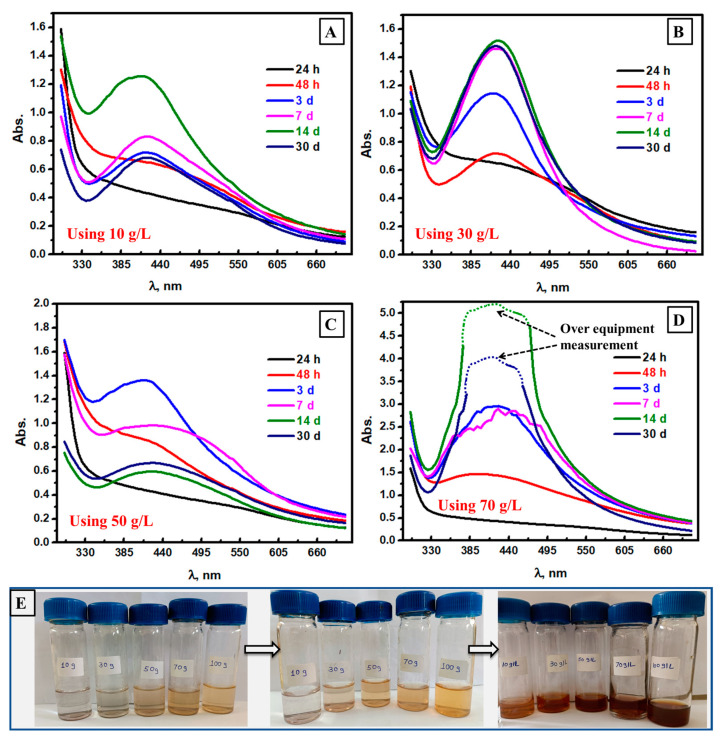
Spectrophotometric study of the formed Ag NPs at different times and using mushroom extract concentrations of 10 g/L (**A**), 30 g/L (**B**), 50 g/L (**C**), and 70 g/L (**D**), and visual photos at different reaction times (**E**).

The effect of mushroom extract concentration was studied, as shown in Figure 1, using different concentrations: 10 g/L, 30 g/L, 50 g/L, and 70 g/L in Figure 4A–D, respectively. As the reaction time went up, the absorbance value increased up to 14 days at 10 g/L mushroom extract, as displayed in Figure 4A. After 14 days, the coagulation of Ag NPs was observed in both visual and spectrophotometric analysis, which could be the main reason for absorbance decay after 14 days [48]. Interestingly, increasing the mushroom concentration extract to 30 g/L could lead to more stability, especially after a longer time (30 days), as shown in Figure 4B. More mushroom contents could lead to faster kinetics, and high absorbance values were seen in the case of 70 g/L (Figure 4D). In particular, the absorbance value increased over equipment measurements at 70 g/L mushroom concentration. The direct visual observation was investigated, as shown in Figure 4E, and agrees with what was seen in the spectrophotometric study. Therefore, the best mushroom extract concentration was found at 30 g/L, as longer time curves were stable compared to other mushroom concentrations.

### 3.5. Characterization of the Synthesized Ag NPs by Edible Macrofungi

**Figure 5:**
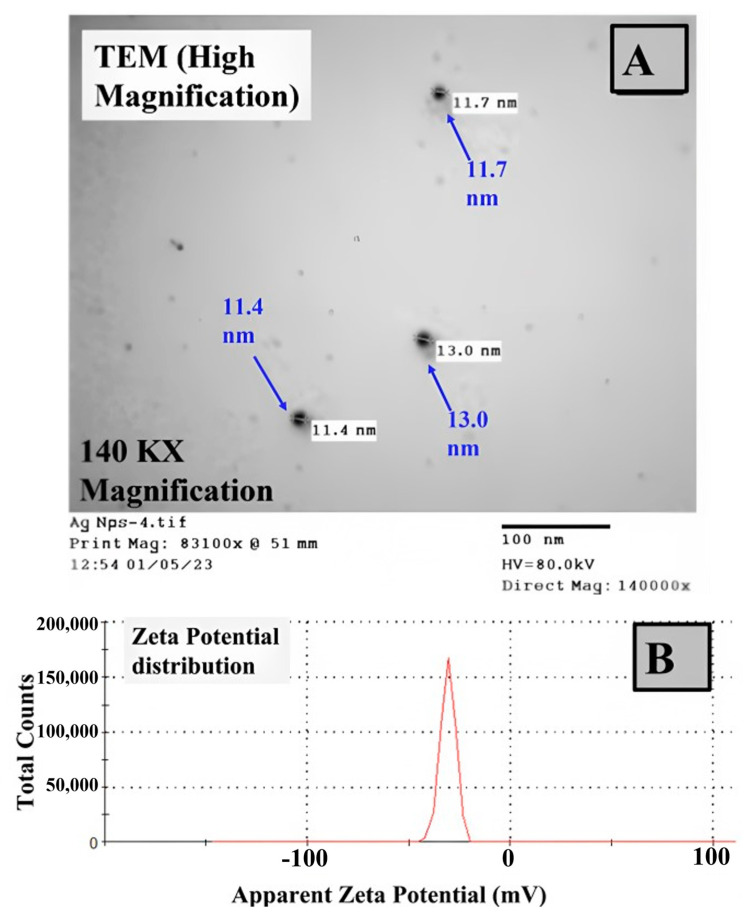
TEM analysis of the formed Ag NPs at magnification: 140 KX (**A**) and Zeta potential analysis of the synthesized Ag NPs using mushroom extract (**B**).

The synthesized Ag NPs from edible mushrooms were characterized by TEM analysis to study morphology. The TEM images were captured at a magnification of 140 KX, as shown in Figure 5A. The morphology of the prepared material is spherical with a nanoscale size of 11–13 nm, which indicates the nano characteristics of the prepared Ag NPs. This result could be due to the use of mushroom extract, which has the characteristics of both a stabilizing agent and a reducing agent, in addition to the reported interaction between protein and silver ions [56,57,58,59]. The stability of the prepared Ag NPs was checked via zeta potential measurements, as displayed in Figure 5B. This investigation could provide information about charges on the nanomaterial surface [60]. Interestingly, the fabricated Ag NPs have an acceptable negative zeta potential of −30.70 mV. This result confirmed the physical and chemical stability of silver NPs suspensions, which could be interpreted by the electrostatic repulsion of the investigated particles [61]. Additionally, the nanoscale-sized particles having negative zeta potential could influence the microbial cells through the possible interaction between their surface and positively charged ions at the cell surface [62]. In short, the edible mushroom was successfully applied to prepare Ag NPs with nano characteristics and negative zeta potential.

**Figure 6:**
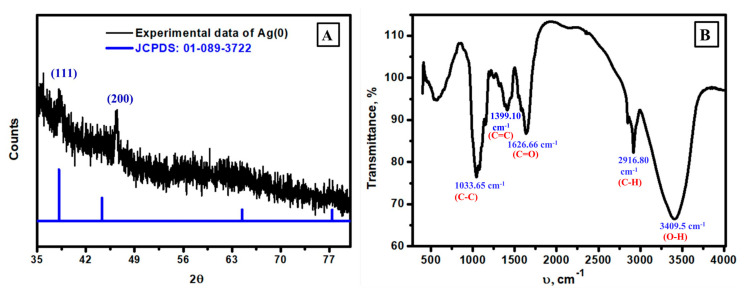
XRD analysis of the formed Ag NPs (**A**) and FT-IR analysis of the synthesized Ag NPs using mushroom extract (**B**).

The crystallinity of the studied Ag NPs was investigated via the XRD pattern, as displayed in Figure 6A. Experimentally, there are two diffraction peaks at 2θ equal to 38.01° and 44.11°, which correspond to crystal planes of (111) and (200), respectively [63]. Additionally, these data are in accordance with the reported JCPDS card no. 01-089-3722 [64], and the crystal structure is cubic with space group fm–3m and space group number 225. The experimental peaks have low-intensity values, which could be due to traces of mushroom content, which could play a considerable role in increasing the amorphous character of the synthesized Ag NPs. The investigation of function groups at the surface of the prepared Ag NPs was studied via FT-IR analysis (Figure 6B) to indicate the chemistry of active functional groups that could play a considerable role during the reduction of Ag ions and their stability after reduction. Figure 6B represents the FT-IR spectrum of the synthesized Ag NPs. The FT-IR diagram clearly shows variable peaks seen at 1033.65 cm^−1^, 1399.10 cm^−1^, 1626.66 cm^−1^, 2916.80 cm^−1^, and 3409.5 cm^−1^ in the studied FT-IR region 400–4000 cm^−1^. These peaks could be interpreted via the vibration of C-C, C=C, C=O, C-H, and O-H, respectively [65,66,67,68], which indicates the existence of traces from mushroom contents at the surface of Ag NPs, which was confirmed via XRD behavior and FT-IR characteristics. To conclude, edible mushroom extraction could reduce silver ions to silver zero valent by interaction with it, and traces of it were found at the surface of cubic Ag NPs, as discussed in XRD and FT-IR analysis.

### 3.6. Antibacterial Activity of the Synthesized Ag NPs by Edible Macrofungi

**Figure 7:**
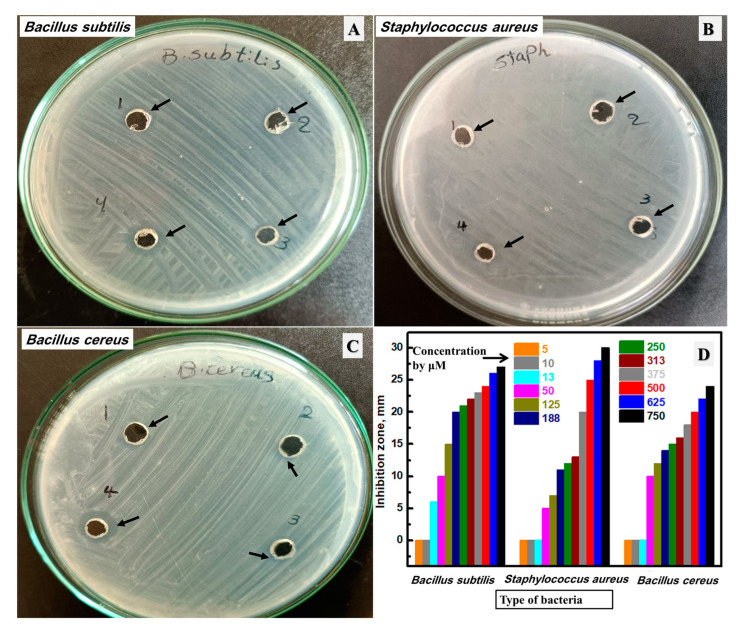
The inhibition zone using the lowest concentrations from the studied Ag NPs of the studied bacterial plates against *Bacillus subtilis* (**A**), *Staphylococcus aureus* (**B**), and *Bacillus cereus* (**C**), and the total investigated concentrations by µM of the synthesized silver NPs (**D**).

The synthesized Ag NPs material was evaluated as an antimicrobial agent against bacteria and fungi. Firstly, the antibacterial activity of the fabricated silver was investigated against seven bacteria. The inhibition zones of the studied bacterial plates against *Bacillus subtilis*, *Staphylococcus aureus*, and *Bacillus cereus* are displayed in Figure 7A–C, respectively. These plates were chosen after testing variable concentrations from 1.25 μM to 187.5 μM, and these plates were selected to have the minimum inhibition concentration (MIC). The inhibition zones of all investigated concentrations were drawn as a bar graph, as described in Figure 7D. The studied Ag NPs have a considerable impact on the bacterial growth of all studied bacteria with different MICs, which was evaluated by measuring the inhibition zones of different concentrations up to the concentration that has no observable zone. Additionally, the increase in the prepared Ag NPs concentration enhanced the inhibition zone at all studied concentrations. The highest-studied concentration (187.5 μM) has an inhibition zone of 27 mm, 30 mm, and 24 mm versus *Bacillus subtilis*, *Staphylococcus aureus*, and *Bacillus cereus*, respectively. Additionally, the lowest-studied concentrations (1.25 μM and 2.5 μM) have no inhibition zone. After that, the concentration of 3.25 μM has an inhibition zone of 6.0 mm against *Bacillus subtilis* and no observable inhibition zone for *Staphylococcus aureus*, or *Bacillus cereus*. Then, the following molar concentration (12.5 µM) has an inhibition zone of 10 mm, 5 mm, and 10 mm versus *Bacillus subtilis*, *Staphylococcus aureus*, and *Bacillus cereus*, respectively. Therefore, the MIC of the fabricated Ag NPs was estimated at 3.25 μM, 12.0 μM, and 12.0 μM against *Bacillus subtilis*, *Staphylococcus aureus*, and *Bacillus cereus*, respectively. Currently, the fabrication of silver NPs is an acceptable way to replace traditional antibiotics for mucosal and skin infections, and so has considerable potential to solve the problem of bacterial resistance [69,70,71,72]. The prepared Ag NPs in this study have a high impact against *Bacillus subtilis*, *Staphylococcus aureus*, and *Bacillus cereus,* which could be attributed to the active surface area that helps silver penetrate or interact with the cell bacterial wall better than bulk antibacterial material and leading at last to bacterial cell death [34,73,74,75]. In short, the prepared Ag NPs material has an excellent inhibition zone against *Bacillus subtilis*, *Staphylococcus aureus*, and *Bacillus cereus* at low concentrations (around 12.5 µM).

**Figure 8:**
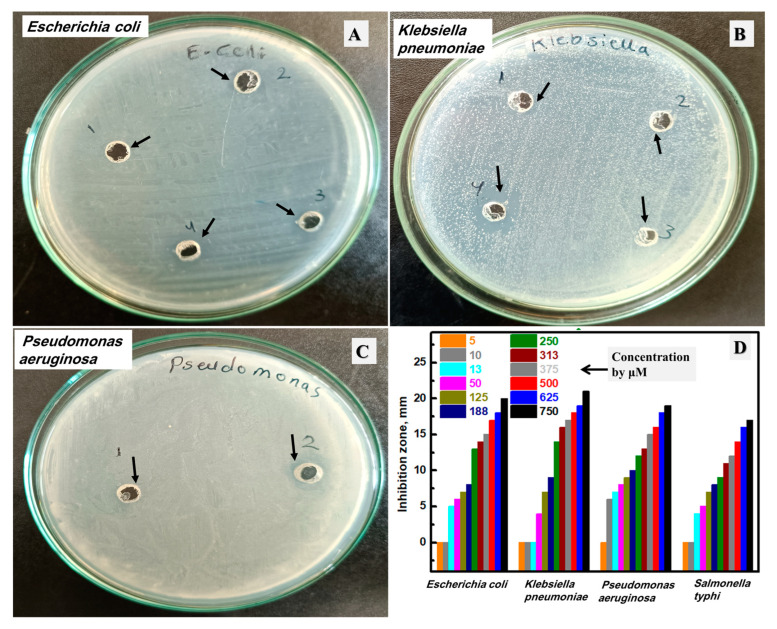
The inhibition zone using the lowest concentrations from the studied Ag NPs of the studied bacterial plates against *Escherichia coli* (**A**), *Klebsiella pneumoniae* (**B**), and *Pseudomonas aeruginosa* (**C**), and the total investigated concentrations by µM of the synthesized silver NPs (**D**).

After that, the synthesized Ag NPs were evaluated as an antibacterial agent against Gram-negative bacteria, including *Escherichia coli*, *Klebsiella pneumoniae*, *Pseudomonas aeruginosa*, and *Salmonella typhi,* as displayed in Figure 8A–C and Figure S3 (Supplementary Material), respectively. These plates were chosen after testing variable concentrations from 1.25 μM to 187.5 μM, and these plates were selected to have the MIC. The inhibition zones of all investigated concentrations were drawn as a bar graph, as described in Figure 8D. The prepared Ag NPs material has a negative impact on the bacterial growth of all studied Gram-negative bacteria with different MICs. Additionally, a higher silver concentration could improve the observed inhibition zone at all investigated concentrations. The highest silver concentration (187.5 μM) has an inhibition zone of 20, 21, 19, and 17 mm against *Escherichia coli*, *Klebsiella pneumoniae*, *Pseudomonas aeruginosa*, and *Salmonella typhi*, respectively. In addition, the lowest silver concentration (1.25 μM) has no inhibition zone. Then, the concentration of 2.50 μM has an inhibition zone of 6.0 mm against *Pseudomonas aeruginosa* and no zone for *Escherichia coli*, *Klebsiella pneumoniae*, or *Salmonella typhi*. After that, the concentration of 3.25 μM has an inhibition zone of 5.0 mm, 7.0 mm, and 4.0 mm against *Escherichia coli*, *Pseudomonas aeruginosa*, and *Salmonella typhi,* respectively, in addition to no observable zone for *Klebsiella pneumoniae*. Then, the next studied concentration (12.5 µM) has an inhibition zone of 6.0, 4.0, 8.0, and 5.0 mm against *Escherichia coli*, *Klebsiella pneumoniae*, *Pseudomonas aeruginosa*, and *Salmonella typhi*, respectively. Thus, the MIC of the biosynthesized silver NPs was estimated at 3.25 μM, 12.5 μM, 2.50 µM, and 3.25 μM against *Escherichia coli*, *Klebsiella pneumoniae*, *Pseudomonas aeruginosa*, and *Salmonella typhi*, respectively. From these results, the prepared silver NPs material in this work has a strong influence against *Escherichia coli*, *Klebsiella pneumoniae*, *Pseudomonas aeruginosa*, and *Salmonella typhi* using the low silver concentration of at least 12.5 µM.

### 3.7. Antifungal Activity of the Synthesized Ag NPs

**Figure 9:**
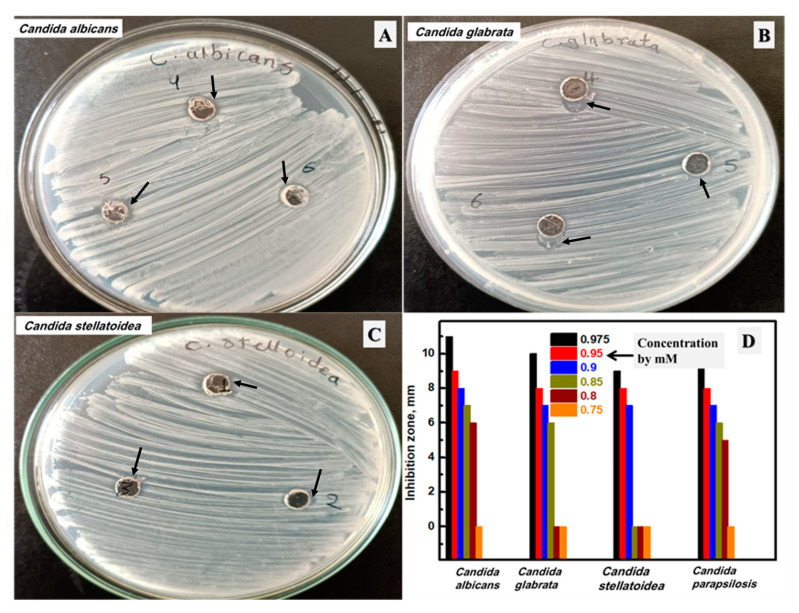
The inhibition zone using the lowest concentrations from the studied Ag NPs of the studied *Candida* plates against *Candida albicans* (**A**), *C. glabrata* (**B**), and *C. stellatoidea* (**C**), and the total investigated concentrations by µM of the synthesized Ag NPs (**D**).

**Figure 10:**
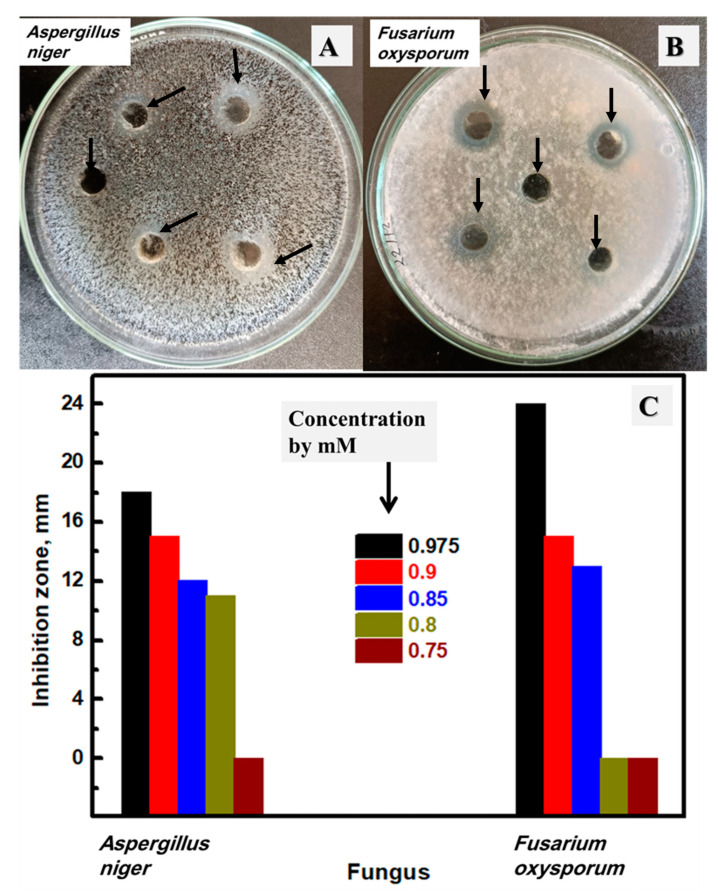
The inhibition zone using the lowest concentrations from the studied Ag NPs of the studied fungi plates against *Aspergillus niger* (**A**), *Fusarium oxysporum* (**B**), and the total investigated concentrations by µM of the synthesized Ag NPs (**C**).

The prepared silver NPs were studied as an antifungal agent against four *Candida species*, including *Candida albicans*, *C. glabrata*, *C. stellatoidea*, and *C. parapsilosis*, as described in Figure 9A–C and Figure S4 (Supplementary Material), respectively, in addition to two fungi, including *Aspergillus niger* and *Fusarium oxysporum,* as shown in Figure 10A,B, respectively. The shown plates were selected after testing variable Ag contents from 750 μM to 975 μM, and these plates were selected to have the MIC value. The measured inhibition zones of all prepared concentrations were drawn, as shown in Figure 9D and Figure 10C for *Candida* and fungi, respectively. The synthesized silver NPs have a negative impact on the *Candida* and fungus growth of all studied microorganisms. In addition, more silver content could enhance the measured inhibition zone at all tested concentrations. The highest silver concentration (975 μM) has an inhibition zone of 11.0, 10.0, 9.0, and 10.0 mm against *Candida albicans*, *C. glabrata*, *C. stellatoidea*, and *C. parapsilosis*, respectively, in addition to 18.0 and 24.0 mm against *Aspergillus niger* and *Fusarium oxysporum*. The lowest silver content (750 μM) has no observed inhibition zone against all studied fungi. After that, the concentration of 800 μM has an inhibition zone of 6.0 mm, 5.0 mm, and 11.0 mm against *Candida albicans*, *C. parapsilosis*, and *Aspergillus niger,* respectively. At this concentration (800 µM), no inhibition zone was detected for *C. glabrata*, *C. stellatoidea*, or *Fusarium oxysporum.* After that, a higher concentration was tested (850 µM), and there was a clear inhibition zone against all studied fungi except *C. stellatoidea*, which started to be inhibited in growth by 900 μM (Its MIC). Therefore, the MIC of the biosynthesized Ag NPs was found at 800 μM, 850 μM, 900 μM, 800 μM, 800 μM, and 850 μM against *Candida albicans*, *C. glabrata, C. stellatoidea*, *C. parapsilosis*, *Aspergillus niger*, and *Fusarium oxysporum*, respectively. Therefore, the prepared Ag NPs stabilized by mushroom extract exhibited considerable antifungal performance as a result of their improved aggregate stability [76,77]. Moreover, the size of NPs could disrupt the yeasts, which improves their sensitivity to Ag NPs [78,79]. Such data and conclusions are in considerable agreement with the previously reported studies introducing the effect of the stabilization of Ag NPs on antimicrobial activity [77,80,81]. In short, the biosynthesized Ag NPs in this work have a considerable influence against different microorganisms using a low Ag concentration of at least 0.8 mM.

## 4. Conclusions

This study presented the biosynthesis of Ag NPs with nanosized particles (11–13 nm), which would play a considerable role in finding novel antimicrobial agents with biocompatible characteristics from the preparation strategy using edible mushrooms. The biosynthesis of Ag NPs was followed by spectrophotometric techniques for optimization of the chemical conditions, including pH, mushroom, and Ag concentration, in addition to the temperature of the mixture during Ag NPs preparation. The results indicate that the best Ag content is 1.0 mM, which has the highest absorbance in the formed spectrophotometric peak analysis. Additionally, the optimum or best pH medium in the case of the biosynthesis of Ag NPs using edible mushrooms is 11, according to the spectrophotometric peak analysis. Regarding mushroom contents, the best mushroom extract concentration was found at 30 g/L, as longer time curves were stable if compared with other studied mushroom concentrations (from 10 g/L to 100 g/L). The Ag concentration of 187.5 μM has an inhibition zone of 27 mm, 30 mm, and 24 mm against *Bacillus subtilis*, *Staphylococcus aureus*, and *Bacillus cereus*, respectively; in addition, an inhibition zone of 20, 21, 19, and 17 mm against *Escherichia coli*, *Klebsiella pneumoniae*, *Pseudomonas aeruginosa*, and *Salmonella typhi*, respectively. The Ag concentration of 975 μM has an inhibition zone of 11.0, 10.0, 9.0, and 10.0 mm against *Candida albicans*, *C. glabrata*, *C. stellatoidea*, and *C. parapsilosis*, respectively, in addition to 18.0 and 24.0 mm against *Aspergillus niger* and *Fusarium oxysporum*. This performance of Ag NPs could be due to the active surface area that helps Ag interact with components of the cell bacterial wall better than the bulk antibacterial agent. To conclude, edible mushrooms could be presented to the biomedical and commercial society to design novel antimicrobial agents with Ag NPs.

## Supplementary Materials

The following supporting information can be downloaded at: https://www.mdpi.com/article/10.3390/biom13071102/s1, Figure S1: Spectrophotometric study of the formed Ag NPs at different pH medium after seven days, Figure S2: Spectrophotometric study of the formed Ag NPs at different times and using mushroom extract concentration of 100 g/L, Figure S3: The inhibition zone using the lowest concentrations from the studied Ag NPs of the studied bacterial plates against *Salmonella typhi*, Figure S4: The inhibition zone using the lowest concentrations from the studied Ag NPs of the studied *Candida* plates against *C. parapsilosis*.
