# Supplementary material for: Biosynthesis, Spectrophotometric Follow-Up, Characterization, and Variable Antimicrobial Activities of Ag Nanoparticles Prepared by Edible Macrofungi
Source: Biomolecules. 2023 Jul 11;13(7):1102. doi: 10.3390/biom13071102 (PMC10377419; doi:10.3390/biom13071102)
Supplement: Supplementary file 1 [file biomolecules-13-01102-s001.zip › biomolecules-2474671-supplementary.pdf]

Supplementary Material

# Biosynthesis, spectrophotometric follow-up, characterization, and variable antimicrobial activities of Ag nanoparticles prepared by edible macrofungi

M. S. Youssef [1], Sanaa Ibrahim Ahmed [1], Ibrahim M. A. Mohamed [2], and Marwa M. Abdel-Kareem [1]

[1] Botany and Microbiology Department, Faculty of Science, Sohag University, Sohag 82524, Egypt.

[2] Chemistry Department, Faculty of Science, Sohag University, Sohag 82524, Egypt.

* Correspondence: ibrahim_mohamed@science.sohag.edu.eg

## 1. Mushroom cultivation:

At first, the mother culture was prepared by cutting small pieces of the gills side from fresh mushroom under sterile conditions. The gills are cultivated in sterile media petri dishes Molded by Media PDA (potato dextrose agar) agar which consists of 200 g potato, 20 g agar, 20 g dextrose, and 1.5 g yeast extract. The potatoes were cut into small pieces and saved in boiling water for 15 minutes followed by filtration. The filtrate was taken and placed with the rest of the media components then, sterilized and poured into sterile Petri dishes. The pre-prepared mushroom parts were cultivated in the obtained

dishes and incubated for 20 days at 25 $^{o}$C. After these 20 days, the mycelium spread all over the plate as shown in Fig. 1. On the other side, wheat grains were soaked in water to remove chaffy and damaged grains. The grains were saved in a vessel for 30 minutes to be softer. Then, the obtained grains were dried at room temperature to remove excess water. The grains were poured in bottles up to 75 % of its height. The obtained bottles were plugged tightly with non-absorbent cotton wool and were covered with aluminum foil. Then, it was put inside an autoclave and sterilized for 2 hours followed by cooling and keeping them inside the culture room. These bottles were injected with pure culture mycelium which was prepared before and then were gently shaken on the 5th and 10th day for distributing the inoculum evenly in the bottles. The process of mushroom cultivation starts with the disinfection of the environment with chlorine to remove traditional pollutants. In this work, different cultivation substrates were used including rice straw, wheat straw, a mixture of rice and wheat straw, a mixture of agricultural lime and wheat straw, a mixture of agricultural lime and rice straw, mixture of quicklime and rice straw. The cultivation steps included the obtained digital photo from the experiments of cultivation. Before cultivation, the seeds were exposed to the air for 12-24 hours in a sterile place. The cultivation substrate was soaked in water for 6 hours then boiling it in water for 1 hour and followed by filtration and cooling at room temperature for 24 hours. The humidity environment was found at 70 to 72% and the environment conditions temperature is from 20-28 C. Mushroom spawn were mixed with cultivation substrate in clear plastic bags, and sealed using adhesive and the formed bags were holed in the edges of the bags to out the excess water. Bags filled with the cultivation substrate and injected with spawn and stored in a dark place away from light, with humidity around 90%, and a

temperature of 24 °C for a period of 15-21 days, which is called the incubation period. The prepared bags and the floor must be sprayed with a water sprayer constantly, in order to provide moisture during the incubation period, and the formation of cotton threads (mycelium). After the incubation period, the second stage started and pale light was applied for 4-6 hours every day away from the sun. Then, the shock was applied by the low thermal stimulus for growth. Mushroom grains begin to appear 15 days after the stimulation process so the moisture of the bags could be maintained, by constantly spraying the bags indirectly with water until the mushroom grains are ready for picking around four days after their appearance so that the first pick is harvested, as there is a period of two weeks between each picking and the other.

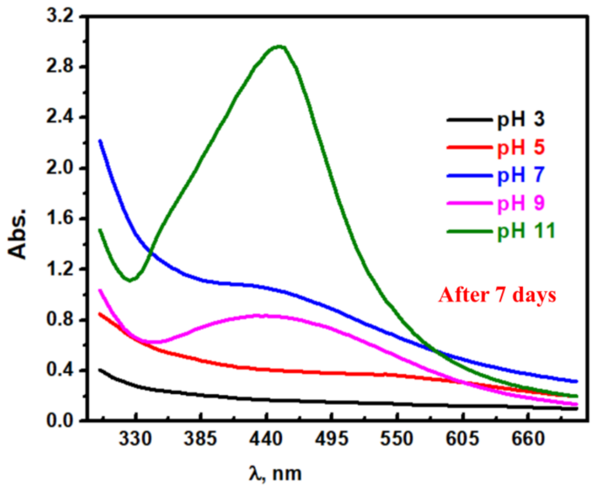


Fig. S1: Spectrophotometric study of the formed Ag NPs at different pH medium after 7 days.

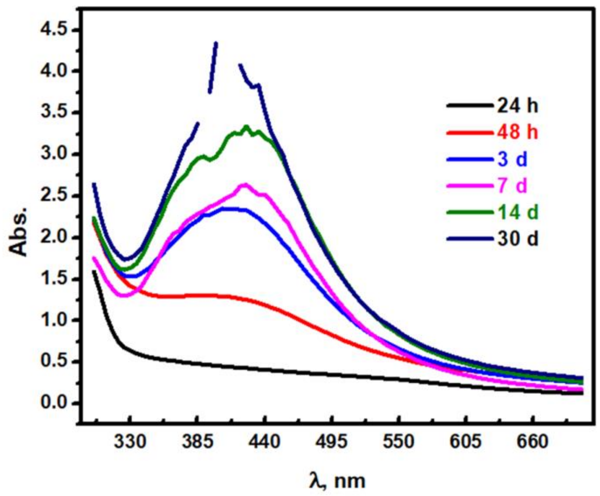


Fig. S2: Spectrophotometric study of the formed Ag NPs at different times and using mushroom extract concentration of 100 g/L

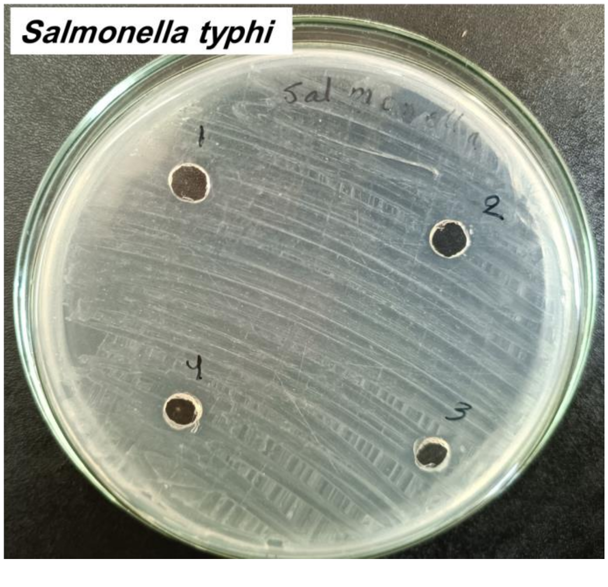


Fig. S3: The inhibition zone using the lowest concentrations from the studied silver material of the studied bacterial plates against *Salmonella typhi*

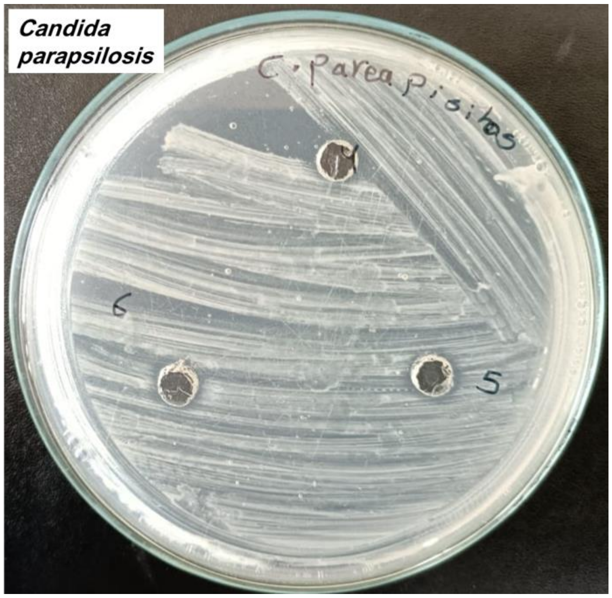


Fig. S4: The inhibition zone using the lowest concentrations from the studied silver material of the studied candida plates against *Candida parapsilosis*
